# PTPN22 Silencing in Human Acute T-Cell Leukemia Cell Line (Jurkat Cell) and its Effect on the Expression of miR-181a and miR-181b

**DOI:** 10.15171/apb.2018.032

**Published:** 2018-06-19

**Authors:** Elham Baghbani, Vahid Khaze, Sanam Sadreddini, Ahad Mokhtarzadeh, Behzad Mansoori, Ali Mohammadi, Vida Vatankhahan, Parisa Toosi, Behzad Baradaran

**Affiliations:** Immunology Research Center, Tabriz University of Medical Sciences, Tabriz, Iran.

**Keywords:** T-ALL, PTPN22, miR-181a, miR-181b

## Abstract

***Purpose:*** T-cell acute lymphoblastic leukemia (T-ALL) is one of the most common malignancies associated with T-lymphocytes, accounting for 10 to 15 percent of ALL cases in children and 25 percent in adults. Innovative therapeutic approaches that overcome ineffective treatments on tumor cells may be a potential source of improvement in therapeutic approaches. Suppression of gene expression at transfusion level is one of the important strategies in gene therapy. The expression of PTPN22 and miR-181 genes in all types of hematologic malignancies increases and is likely to contribute to the survival and death of cells by affecting a variety of signaling pathways. The purpose of this study was to determine the role of PTPN22 inhibition by siRNA, and alteration in miR-181a and miR-181b in Jurkat cell line.

***Methods:*** Jurkat cells were transfected with 80 pmol of siRNA to inhibit PTPN22. After that, expression of PTPN22 mRNA and transcript levels of miR-181a and miR-181b were measured with Real-time PCR after 48hrs.

***Results:*** Experiments demonstrated that siRNA transfection resulted in significant downregulation of PTPN22 mRNA after 48 hrs in 80 pmol dose of siRNA. Moreover, transcript levels of both miR-181a and miR-181b was decreased after transfection.

***Conclusion:*** PTPN22, miR-181a and miR-181b might be involved in progression of Jurkat cells and targeting these molecules by RNAi might confer promising tool in treatment of T-ALL.

## Introduction

T-cell acute lymphoblastic leukemia (T-ALL) is considered as one of the most frequent malignancies associated to T-cells, which is typically reflected as an invasive tumor.^1^ Overall, over 80% of patients reply to chemotherapy with complete clinical remission, and up to 50% experience relapse with chemoresistant disease.^2^ Despite a powerful 40%–50% overall survival rate (reached over the past ten years), refractory relapsed leukemia remains an unsolved therapeutic problem.^3,4^ Therefore, therapies with another mechanism of action are instantly required. Effectiveness of RNA interference (RNAi) in cancer medical care has been characterized by high efficiency and potential, induction of silencing within the advanced stages of growth, transmission of silenced gene to consequent generation, low price compared to the opposite strategies of gene therapy, and high specificity compared to the opposite strategies of cancer therapy like chemotherapy and lack of side effects compared to chemotherapies.^5,6^

*Protein tyrosine phosphatase, non-receptor type 22* (*PTPN22*) gene is located on human chromosome1p13.3-p13.1 and encodes a protein referred to as lymphoid tyrosine phosphatase (Lyp) in human and PEST-enriched protein tyrosinephosphatase (Pep) in mice.^7^ PTPN22 is expressed in T, B, NK, and dendritic cells. The physiological function of PTPN22 has been studied primarily in the context of T cells; however, it is not fully understood. PTPN22 is supposed to be located in the cytoplasm of T-cells and interacts with many signaling molecules, such as Lck, ZAP70, Csk, and Vav, thereby attenuating TCR signals.^8^ Juxtaposition of promoter and enhancer components of TCR genes with transcription factor genes throughout VDJ recombination is among the cytogenetic changes inflicting T-ALL. Cytogenetic changes play a crucial role in leukemogenesis in cancers of immune cells together with T-ALL by altering the expression and function of miRNA, which may perform as tumor suppressors or oncogenes.^9^

MicroRNAs (miRNAs) are single stranded ~22 nucleotides (nt) long non-coding RNAs that regulate gene expression at the post-transcriptional level. MiRNAs are encoded in host genes, which may be placed in introns or exons of protein-coding genes, also as in non-coding genes.^10^ miRNA restrictive roles in numerous biological process and physiological events, and disease pathological process have become evident within the previous few years.^11^ To date, solely a number of studies has addressed the importance of miRNAs in haematopoiesis.^12^ The expression profiles of murine hematopoietic-specific miRNAs (miR-142, miR-181 and miR-223) are represented in B and T cells, monocytes, granulocytes, and erythroid cells.^13,14^

MiR-181 was concerned in regulation of the differentiation of B cells, T cells and natural killer (NK) cells during normal hemogenesis. Its family has four members (miR-181a, miR-181b, miR-181c, and miR-181d).^15^ It has additionally been noted that miR-181 includes a dual behavior and acts as a tumor suppressor in glioma,^16^ and in oral epithelial cell carcinoma,^17^ however, it also behaves as an onco-miRNA in non-small-cell lung cancer,^18^ breast cancer,^19^ hepatocellular carcinoma,^20^ gastric and colon cancer.^21^ Additionally, miR-181a was very powerfully expressed within the thymus, the primary lymphoid organ, that chiefly contains T lymphocytes. MiRNA-181a plays a crucial role within the selection and activation of T cells, and miRNA-181a deficiency results in the rising the rate of autoreactive lymphocytes. PTPN22 and DUSP6 (both have a key role throughout T cell activation) are targets of miRNA-181a.^22^ Accumulating evidence indicates that miR-181a incorporates a role in the development of medical specialty malignancies; however, it is still unclear. Some findings suggest that miR-181a behaves as an onco-miRNA in leukemia. Other indicates miR-181a acts as a tumor suppressor.^23^

Oncomirs, mutated tumor suppressor genes, and a number of other genes concerned in tumor progression are smart targets for gene silencing by RNAi-based therapy.^24^ The most important advantage of RNAi in cancer therapy is targeting multiple genes of assorted cellular pathways concerned in tumor progression.^25^ Here, we specifically investigated the effects of PTPN22 silencing in human acute T-cell leukemia cell line (Jurkat cell) and its effect on the expression of miR181a and miR181b. We have shown that PTPN22 can be considered as a potent target molecule in T-ALL therapy.

## Materials and Methods

### Cell culture

RPMI-1640 culture medium and fetal bovine serum (FBS) were purchased from GibcoBRL Company. Jurkat cell line was obtained from the Iranian Biological Research Center (IBRC), Tehran, Iran. Jurkat cells were grown in RPMI-1640 supplemented with 10% (v/v) FBS, penicillin (100 U/ml), streptomycin (100 µg /ml; SimgaAlderich, St. Louis, MO, USA), and maintained at 37 C in humidified 5% CO_2_ atmosphere.

### siRNA transfection

siRNA was purchased from Santa Cruz Biotechnology. Before siRNA transfection, the Jurkat cells were seeded at a density of 2x10^5^ cells/well in 6-well plates (untreated cells were kept as controls) in serum and antibiotic free RPMI-1640 medium. All transfections were performed using siRNA according to the manufacturer’s recommendations. In brief, transfection reagent (6 µl/ml of transfection reagent) and siRNA (at a final concentration of 80 pmol) were diluted in siRNA transfection medium (Santa Cruz Biotechnology) separately and mixed gently. After incubation for 20 min at ambient temperature, the diluted solutions were combined and incubated for another 30 min at ambient temperature. Thereafter, the mixtures were added to each well (the cells incubated only with transfection reagent were considered as controls). The cell culture plates were then incubated for 6 hrs at 37 °C in a CO_2_ incubator. The cells incubated under the aforementioned conditions were added in RPMI-1640 medium containing FBS and antibiotics (concentration of 2 normal growth medium) without removing the transfection mixture.

### qRT-PCR

For PTPN22 transcription evaluation, total RNA was isolated from cells using RiboEx reagent (GENEALL Biotechnology, Seoul, Korea) according to the manufacturer’s instructions. The reverse transcription reactions were performed with randomhexamer primer and a Reverse Transcriptase M-MLV (Takara, Korea) following the manufacturer’s protocol. Quantitative Real-time PCR was performed using a standard SYBR Green PCR master mix (Takara, Korea) protocol on a Roche LightCycler 96 system (Roche, Germany) according to the instructions.

β-actin was used as reference gene for determining the mRNA expression level of PTPN22. The primer sequences are listed in Table 1. Each sample was analyzed in triplicate. The 2^-ΔΔCt^ method was used to determine the relative quantitation of gene expression levels.^26^

To determine the transcription levels of miR181a and miR181b, total RNA was isolated from cells using RiboEx reagent (GENEALL Biotechnology, Seoul, Korea) according to the manufacturer’s manuals. Complementary DNA (cDNA) was synthesized by cDNA Synthesis Bioneer kit from 1 mg of total RNA. Following this, qRTPCR was performed in the Roche LightCycler 96 system (Roche, Germany). The PCR reaction conditions were as follows: 3 µl of cDNA template, 0.5 mM of each primer, 12 µl of SYBR green reagent, and 9 µl of nuclease-free distilled water. The cycling conditions were as follows: 94 °C for 5 min for cDNA and primer denaturing, followed by 35 cycles at 94 °C for 30 s, 56 °C for 30 s, and 72 °C for 40 s. Quantitative RT-PCR was carried out for miR-181a and miR181b in total volume of 10 μl reaction mixture using miRCURY LNA™ Universal RT microRNA PCR and SYBR Green master mix (Exiqon, Vedbæk Denmark) according to the manufacturer’s protocol. Amplification was performed as follows: 95 °C for 10 min, 40 cycles of 95 °C for 10 s and 60 °C for 10 s, ramp rate 100% under standard condition. miR-U6 was used as the housekeeping gene. The primer sequences were as follows in Table 1. Each sample was analyzed in triplicate. The relative quantitation of gene expression levels was calculated using 2^-ΔΔCt^ method.^26^

**Table 1:** Primer sequences in Real-time PCR.

**Target gene**	**Strand**	**Primer sequence**
β-actin	Forward	5ʹ-TCCCTG GAGAAGAGCTACG-3ʹ
	Reverse	5ʹ-GTAGTTTCGTGGATGCCACA-3ʹ
PTPN22	Forward	5ʹ-CCAGCTACATCAATGCCAACTTC-3ʹ
	Reverse	5ʹ-CCAAATCATCCTCCAGAAGTCC-3ʹ
miR-181a	Target sequence	5ʹ-AACAUUCAACGCUGUCGGUGAGU-3ʹ
miR-181b	Target sequence	5ʹ-AACAUUCAUUGCUGUCGGUGGGU-3ʹ

### Statistical analysis

All data were analyzed using Graphpad Prism software version 7.0 (Graph Pad Prism; San Diego, CA, USA) and was expressed as means ± standard deviation (SD). The Kolmogorov-Smirnov test was conducted to evaluate the normality of the scale variables. Mann-Whitney *U* and Kruskal-Wallis tests were used for comparing groups with parametric data. *P*<0.05 was considered to indicate a statistically significant difference.

## Results and Discussion

### siRNA downregulated PTPN22 mRNA in Jurkat cells

**Figure 1:**
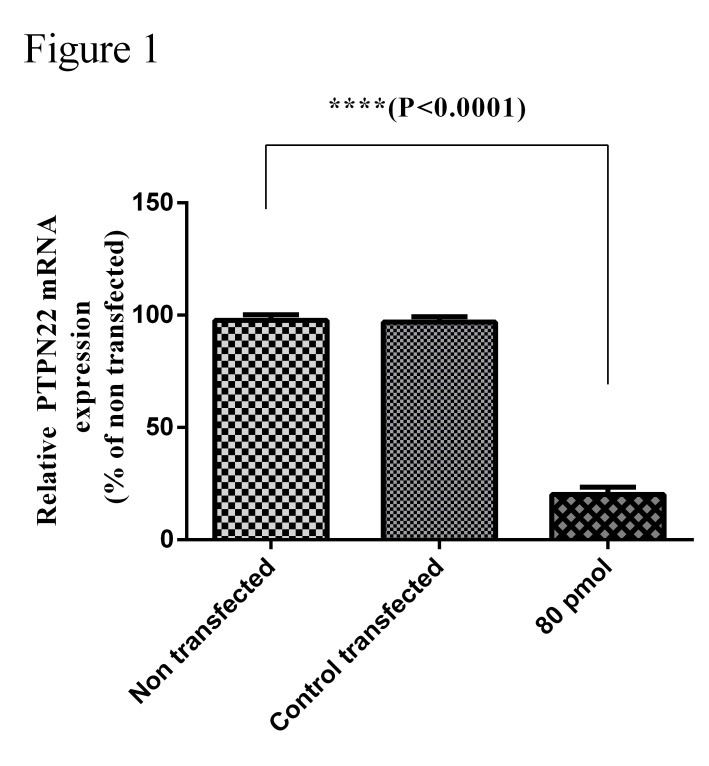
**Inhibition of PTPN22 gene in the Jurkat cell line.** Cells were transfected with siRNA specific to 80 pmol of PTPN22 and 48hrs after transfection, the RNA of the cells was extracted and the expression of the PTPN22 mRNA was evaluated by qRT-PCR (P<0.0001).

The results showed that specific siRNA transfection downregulated significantly (*P*<0.0001) the expression of PTPN22 mRNA compared with the control group. At 48 hrs after transfection with 80 pmol of the specific siRNA molecule of PTPN22, the expression of PTPN22 was 23% (Figure 1).The results showed that highest reduction in expression level of the PTPN22 mRNA in Jurkat cells treated by specific siRNA was achieved at a dose of 80 pmol and 48 hours after transfection.

### Downregulation of PTPN22 lead to downregulation of miR-181a 

**Figure 2:**
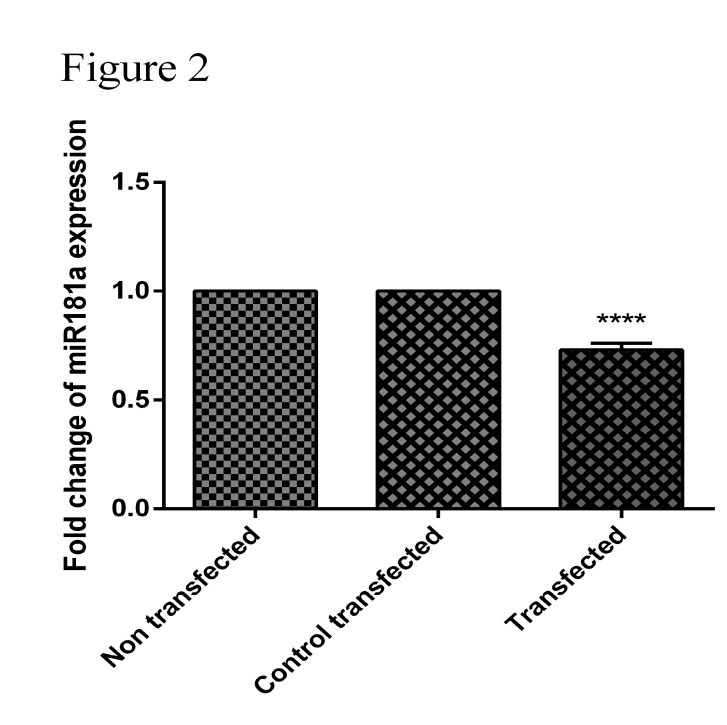
**Effect of PTPN22-siRNA on miR-181a expressions in Jurkat cell line.** After transfection with 80 pmol of PTPN22-siRNA and 48 hrs after transfection, RNA was extracted and the expression of miR-181a was detected by qRT-PCR. **** indicates a significantly decrease in miR-181a levels in Jurkat cells following transfection with PTPN22-siRNA (80pmol dose) compared with the control group cells (P <0.001).

The expression of miR-181a (Figure 2) in Jurkat cells after transfection in a dose of 80 pmol of siRNA-specific PTPN22 was evaluated by qRT-PCR assay. The results showed that the miR-181a expression levels was decreased significantly after transfection with liposomes containing PTPN22 specific siRNA in Jurkat cells (*P*<0.05).

### Downregulation of PTPN22 lead to downregulation of miR-181b

**Figure 3:**
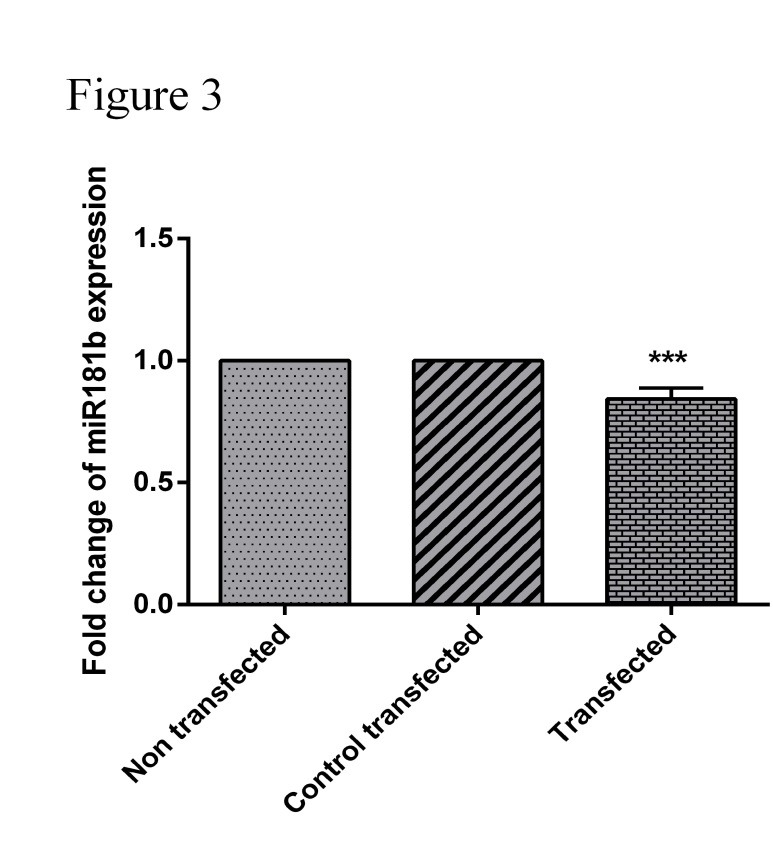
**Effect of PTPN22-siRNA on miR-181b expressions in Jurkat cell line.** After transfection with 80 pmol of PTPN22-siRNA and 48 hrs after transfection, RNA was extracted and the expression of miR-181a was detected by qRT-PCR. *** indicates a significantly decrease in miR-181b levels in Jurkat cells following transfection with PTPN22-siRNA (80 pmol dose) compared with the control group cells (P <0.0001).

The expression of miR-181b (Figure 3) in Jurkat cells after transfection in a dose of 80 pmol of siRNA-specific PTPN22 was evaluated by qRT-PCR assay. The results showed that the miR-181b expression levels was decreased significantly after transfection with liposomes containing PTPN22 specific siRNA in Jurkat cells (*P*<0.05).

Despite significant advances in immunological treatment and diagnosis of T-cell acute lymphoblastic leukemia (T-ALL), the disease is associated with high mortality rate in adults. The therapeutic efficacy has been reported to be between 25% and 40%.^27^ Better understanding of the mechanisms of molecular biology of leukemia can provide a better knowledge of their specific pathogenicity mechanisms and better therapeutic strategies.^28^

Nowadays, targeted molecular therapy is a growing new technology for cancer treatment. A strong approach of gene therapy is a targeted treatment, in which the target is a specific gene that is enhanced or suppressed in tumor cells.^29^ One of the therapeutic factors in this method is exertion of siRNA, which is used to interfere with the expression of the desired gene attributed to the formation, growth, and metastasis of the tumor. The use of siRNA is a strong approach to inhibit specific genes.^30,31^

More than half of the known miRNAs are located in genomics are associated with cancer,^32^ which indicate the important role of miRNAs in all stages of the cancer. Although understanding how miRNAs affect the pathogenesis of cancer is complicated, studies have shown that the abnormal expression of miRNAs can be considered as biomarkers for the prognosis, diagnosis, classification, and treatment of cancer.^10^ Each tissue expresses a different level of specific miRNAs and in fact has a specific expression pattern. It is, therefore, natural that any tumor also has a specific expression pattern of miRNAs, which can be used to detect the origin of metastatic tumors and to distinguish between different subtypes of a specific cancer.^33^ Even this particular pattern can be related to the degree of tumor and status of patient. Increasing evidence suggests that miRNAs play an important role in cancer biology, and recent studies have established the role of oncogenic and tumor inhibitory miRNAs in cancer cells.^34,35^

Hyperactivity of PTPN22 can disrupt the function of T lymphocytes.^36^ Impaired activity of these cells is due to a disruption of the evolution of the intracellular messaging process, which interferes with host immune responses.^7^ It seems that the lack of balance of humoral and cellular immune responses due to impaired PTPN22 function plays a role in the pathogenesis of autoimmune diseases such as rheumatoid arthritis and multiple sclerosis.^37^ On the other hand, studies have also demonstrated the role of PTPN22 in pathogenesis of lymphoid and myeloid leukemia.^38^

It has been shown that the miR-181 family plays an important role in mechanobiology of blood cells, including lymphocytes and monocytes. Moreover, miR-181a plays an important role in maturation and differentiation and function of T lymphocytes. Aberrant expression of miR-181a has been observed in a variety of malignancies, including breast cancer, liver, colon, and leukemia.^39,40^

In this study, we examined the effect of siRNA transfection on the expression of PTPN22 gene and its consequence on miR-181a and miR-181b in Jurkat cell line. In our previous study, we investigated the role of PTPN22 in acute lymphoblastic T-cell leukemia and showed that this gene contributed to the increased survival and proliferation of cancer cells of acute T cell lymphoblastic leukemia.^41^ In this study, it was observed that treatment with PTPN22-siRNA resulted in decreased expression of PTPN22 at the level of mRNA after 48 hours in Jurkat cells. Moreover, the highest efficiency in suppression was obtained at a concentration of 80 μM. Furthermore, the results indicated that the expression levels of miR-181a and miR-181a were significantly reduced after transfection in Jurkat cells.

## Conclusion

Considering all the facts, we indicated that the specific suppression of PTPN22 by siRNA significantly reduced the expression of this gene in Jurkat cells. In addition, suppression of PTPN22 gene expression led to downregulation of miR-1811 and miR-181b. As a result, PTPN22 and miR-1811 and miR-181b could serve as factors in designing therapies for T-ALL as well as biomarkers for diagnosis of this cancer.

## Acknowledgments

This work was performed at the Immunology Research Center (IRC), Tabriz University of Medical Sciences. We thank staff of the IRC for their technical assistance.

## Ethical Issues

Not applicable.

## Conflict of Interest

The authors report no declarations of interest.
